# Autonomy for MRI Field Cameras: Synchronization, Self‐Calibration, and Sequence Detection

**DOI:** 10.1002/mrm.70339

**Published:** 2026-04-02

**Authors:** Oskar Björkqvist, Klaas P. Pruessmann

**Affiliations:** ^1^ Institute for Biomedical Engineering ETH Zurich and University of Zurich Zurich Switzerland

**Keywords:** autonomy, field cameras, field probes, position calibration, sequence measurement

## Abstract

**Purpose:**

This work aims to provide a workflow for operating NMR field probes in MR scanners independently of pulse programming, scanner TTL trigger pulses and dedicated calibration sequences.

**Methods:**

An independent timing system and limited prior knowledge about relative probe positions enable synchronous field measurement, probe position calibration, and sequence parsing.

**Results:**

The proposed procedures were implemented and tested by measurement in a 3 T MRI system. They were found to achieve field‐probe and field‐camera functionality fully independently, without connection to or communication with the scanner. Autonomous position calibration deviated from the conventional method by less than 300 μm. Self‐synchronization permitted the recording of sequence schematics and targeted measurement of sequence features of interest.

**Conclusion:**

Magnetic field sensing in MRI systems can be rendered autonomous and self‐reliant for independent measurement. This capability paves the way for platform‐agnostic field cameras and could readily integrate with existing systems.

## 1 Introduction

Magnetic resonance imaging relies on generating magnetic fields with very high fidelity in both space and time. However, fields and field dynamics are realized only with limited accuracy due to eddy currents, vibration, drift, heating, and gradient amplifier limitations, among others [1]. Remaining imperfections call for direct spatiotemporal measurement of magnetic fields [2, 3, 4]. Several mechanisms are suited to this task, including inductive sensing [5], the use of the Hall effect [6], and atomic magnetometry [7]. However, the currently preferred means of field sensing in MRI systems is NMR itself, offering superior sensitivity in high background fields. Field measurement based on NMR probes has become well established in MRI research in the past decade. Such NMR probes are available in field camera arrangements [8] and also as clip‐on probes which can be mounted in spatially constrained settings. Field cameras are used for hardware characterization, image reconstruction, as well as sequence development and verification, elucidating encoding mechanisms [9, 10, 11, 12, 13, 14]. One disadvantage of NMR field probes is that they suffer from limited signal duration, a property innate to pulsed NMR that precludes continuous field measurement. Due to their non‐continuous nature, targeted NMR field probe measurements must be coordinated with scanner operation, requiring the modification of sequences to produce appropriate triggers. In the same vein, custom sequences are also necessary for position calibration, that is, the initial determination of exact probe positions. These features are not usually available on product MRI systems. Modification of scanner software requires authorized access and platform expertise and is thus often not an option.

The need to explicitly coordinate between scanners and field cameras is also unsatisfactory conceptually. A measurement device should inherently be independent of the respective system under study or device under test.

The goal of the present work is to achieve independence and autonomy for field cameras, along with the ability to fully capture MRI sequences despite finite signal lifetimes. We demonstrate self‐calibration and self‐synchronization, the generation of gradient sequence diagrams, and targeted measurement of features of particular interest, all completely independently of the scanner under study.

## 2 Methods

### 2.1 System Overview and Workflow

**FIGURE 1:**
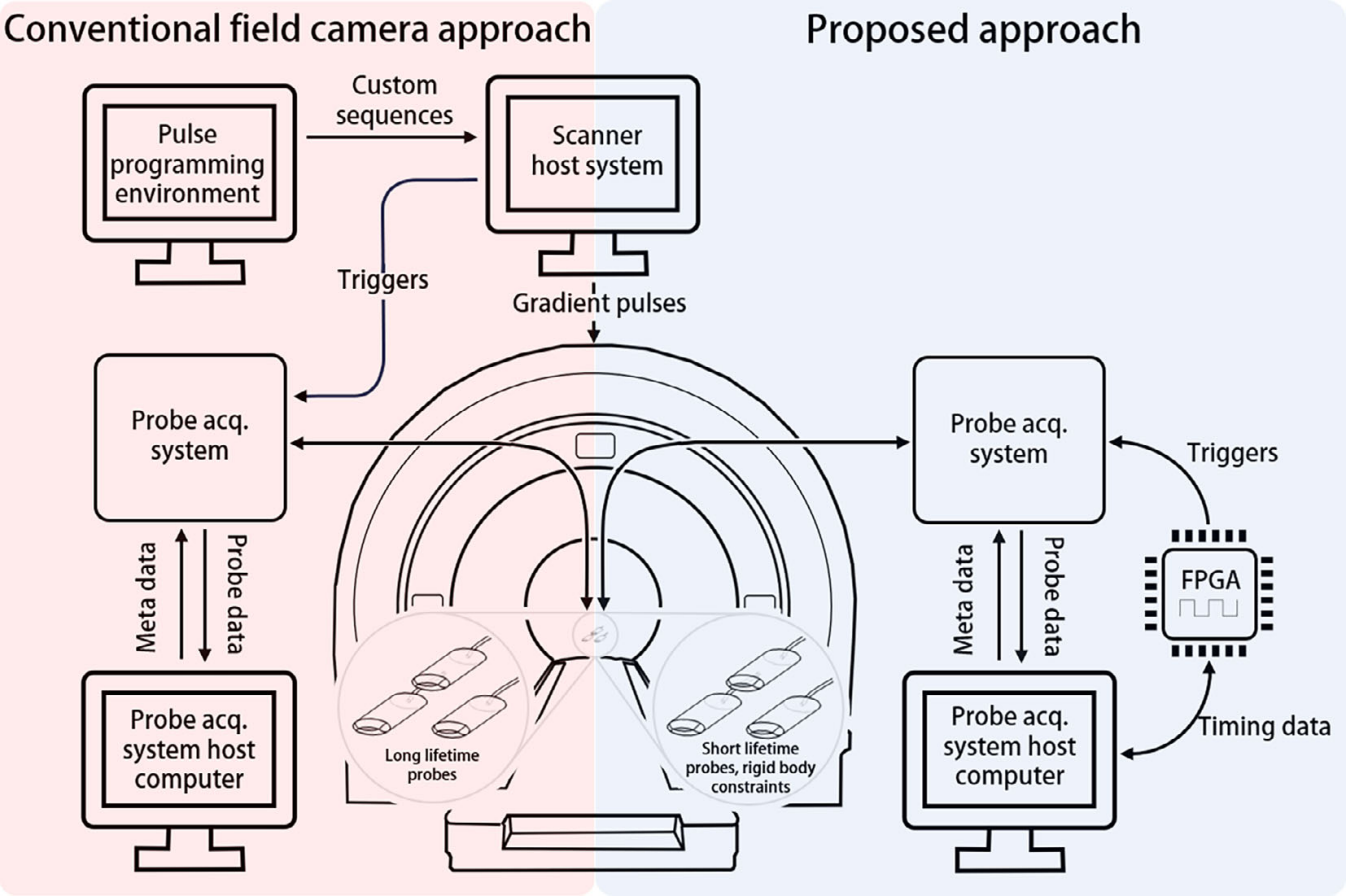
Overview of a conventional field camera and its workflow (left) and our proposed approach (right). The most notable differences between the two are the absence of communication between the probe acquisition system and scanner and operation of the field camera without any software or sequence modifications on the scanner.

To perform field measurements in an MRI system, a set of NMR probes are placed in its imaging volume. Probes made for MRI uses typically have long relaxation times (*T*_1_ ≈100 ms) as a way to maximize the possible continuous measurement time. These probes can be clip‐on, assembled in a camera [8], or integrated, for example, in a head coil [10, 15, 16, 17, 18, 19], and are connected to an acquisition system responsible for excitation and measurement. Such a setup conventionally relies on customization of imaging sequences, particularly for position calibration and the output of triggers to synchronize probe operation. This conventional setup is illustrated on the left‐hand side of Figure 1.

In our proposed system, on the right‐hand side, connections to the scanner system are removed. This is made possible by use of a Field Programmable Gate Array (FPGA) as an external timing and triggering device, which communicates timing information to and from the probe host computer and sends measurement triggers to the probe acquisition system. This setup no longer relies on customized sequences but self‐synchronizes and uses generic sequences for calibration. We choose to work with probes with shorter relaxation times (*T*_1_ < 10 ms) as a way of enabling rapid re‐excitation when probes dephase [20]. The probes have a 1.3 mm sample diameter and rely on ^19^F NMR in hexafluorobenzene doped with Gd(FOD)_3_ [21]. The probes are of clip‐on type and are deployed in one or multiple rigid‐body configurations with known pairwise distances.

**FIGURE 2:**
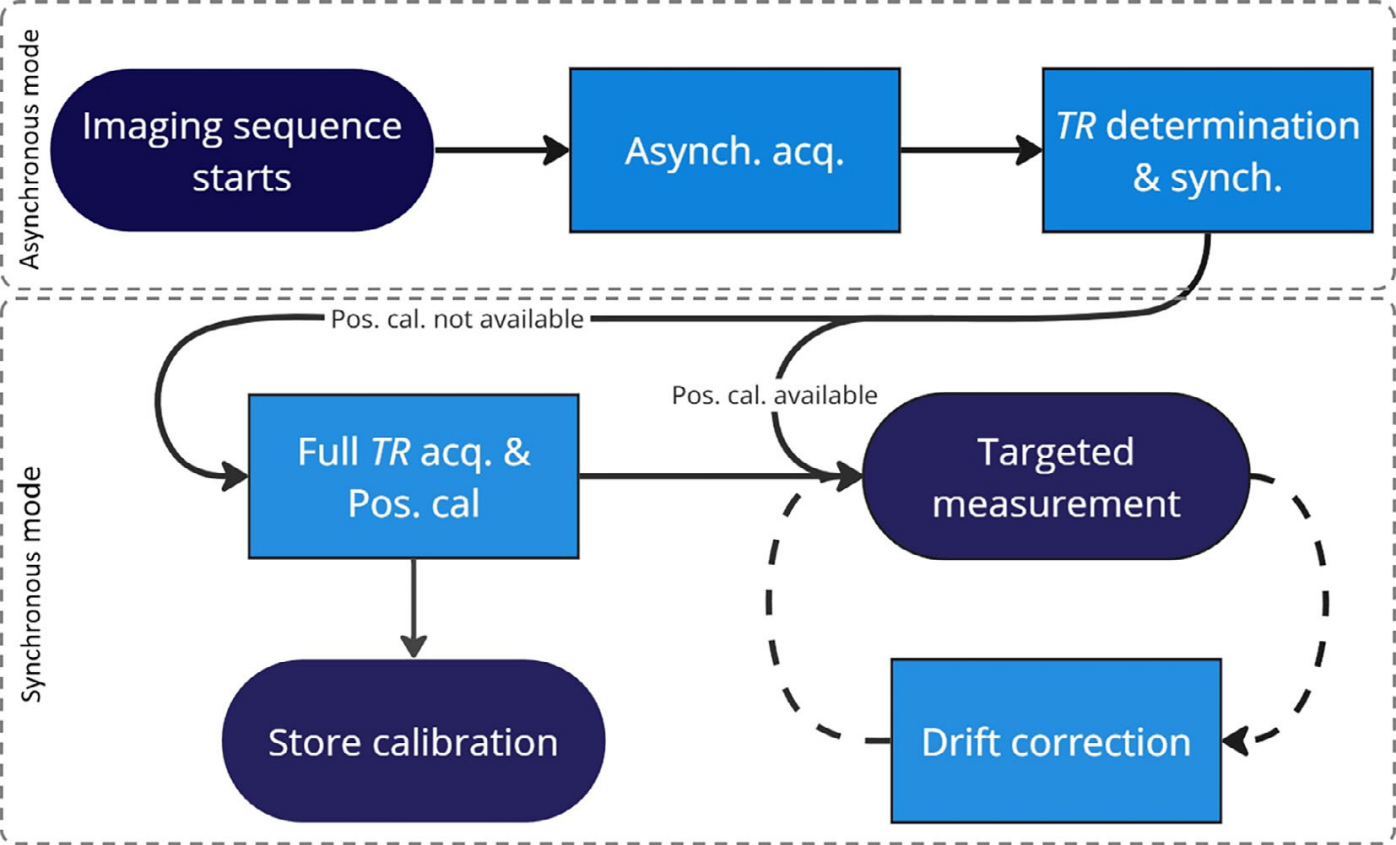
Workflow for our proposed method: An initial, asynchronous phase is always required to achieve a shared sense of time between the sequence and the system timing. Once established, measurements made during the asynchronous phase can be put into the context of the sequence timing and targeted measurements can be made.

The workflow for the proposed system is illustrated in Figure 2. The first step is establishing a shared sense of time between the system and a sequence running on the scanner. This is achieved by asynchronous field probe measurements, which allow detection of the sequence's periodicity. Once the period is found, past and future measurements can be placed in the sequence timing context, effectively synchronizing the probe system with the scanner. After synchronization, probe positions are determined using generic sequence features and knowledge of probe distances. The resulting position calibration remains valid for observing arbitrary subsequent sequences and other field dynamics. Self‐synchronization based on asynchronous acquisition is repeated each time a sequence is started on the scanner. During a scan, timing drift correction may become necessary due to finite precision of periodicity detection and because the FPGA and scanner clocks are not locked.

These procedures replace the calibration and synchronization workflow of conventional field cameras, obviating the need for any connection to the MRI system under study and modification of its software. In the following sections, we detail each of the steps in the flowchart in Figure 2 and show how they are implemented.

### 2.2 Synchronization

Because the FPGA clock is separate from sequence timing, a relationship must be established between them. This is possible because most MR sequences exhibit periodic features. For example, typical spin‐warp sequences have regularly repeating RF pulses and slice‐select and readout gradients. Once this periodicity is identified, a cyclic counter on the FPGA can be aligned with the running sequence.

**FIGURE 3:**
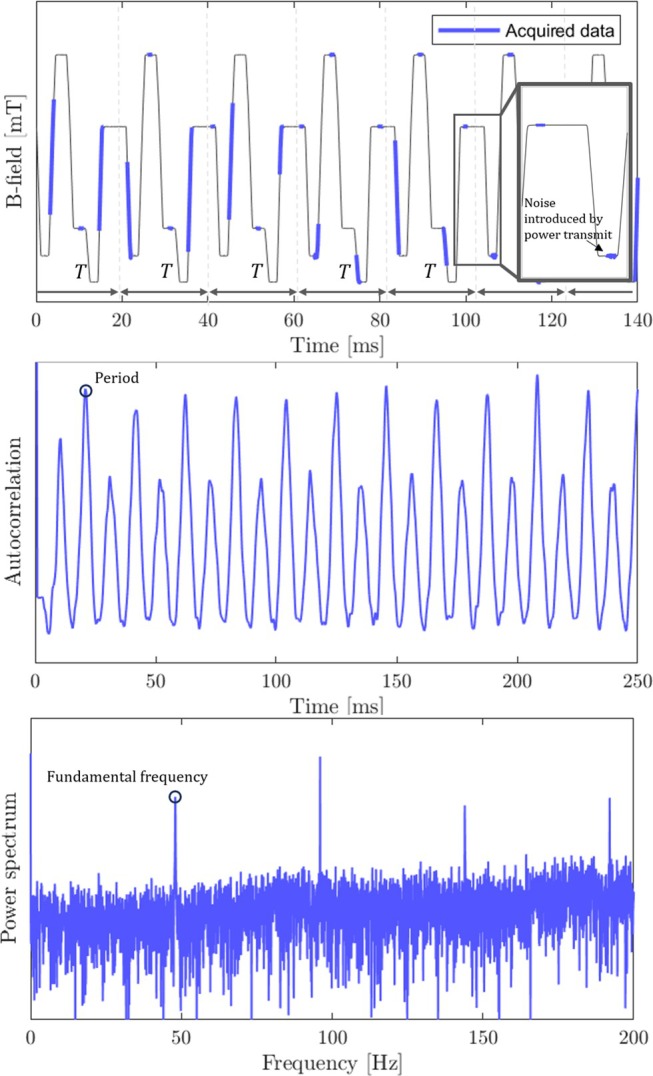
*Top*: Asynchronous field measurement: Randomly timed readout snippets from one probe overlaid on the underlying field time course. *Middle*: Autocorrelation of the asynchronous signal. *Bottom*: Power spectrum of the asynchronous signal.

Initially, all measurements are referenced only to the FPGA clock. A first asynchronous set of acquisitions is made over multiple sequence periods: each measurement lasts about 1 ms and is randomly timed. The measurement snippets are immediately differentiated to represent field rather than accrued phase. This produces an intermittent field time course from each probe (top row of Figure 3), from which periodicity must be extracted. The use of random undersampling to identify sparse spectral support is closely related to compressed sensing [22]. Finding a fundamental frequency is also common in audio processing [23, 24], where spectral methods are often combined with autocorrelation, with periodic peaks representing the fundamental frequency (Figure 3, middle row). We also compute the power spectrum, which typically exhibits a first prominent peak at the fundamental frequency. The prominence of these markers depends on the duration of the underlying asynchronous acquisition, which is thus continued until the fundamental frequency is unequivocal. Once the periodicity is found in this way, it is refined using epoch folding [25, 26] where data are divided into segments of a given trial period to reveal periodic signals. To do this, probe data is stacked in a matrix, which we will refer to as an epoch matrix, with width corresponding to a putative period *T* and height to the number of periods covered in the measurement:

(1) $$d^{\left(p\right)}=\left[\begin{array}{ccc} d_{1,1} & \cdots & d_{1,N_{T}}\\ d_{2,1} & \cdots & d_{2,N_{T}}\\ d_{3,1} & \cdots & d_{3,N_{T}}\\ \vdots & \vdots & \vdots \end{array}\right]$$

where *p* is the probe index. If the trial period *T* is not a multiple of the sampling interval, the data is interpolated such that each column corresponds to a specific point within the proposed period. *T* is then varied until observations from different periods overlap, minimizing the cost function

(2) $$L\left(N_{T}\right)=\sum_{p}^{N_{P}}\sum_{t}^{N_{T}}\left|\max\left(d_{:,t}^{\left(p\right)}\right)-\min\left(d_{:,t}^{\left(p\right)}\right)\right|$$

This approach relies on sufficient coverage of the period: the argument $|\max\left(d_{:,t}^{\left(p\right)}\right)-\min\left(d_{:,t}^{\left(p\right)}\right)|$ only yields a meaningful result if a point within the period has been observed at least twice. Modeling the required observation time is analogous to the random arc covering problem, which has been studied extensively in the probability literature [27]. In practice, the method requires several tens of observation periods in lab time to determine the period.

If the initial estimate of T is sufficiently accurate, gradient descent can be applied to avoid exhaustive search. While the method can operate with a single probe for faster computation, robustness and precision improve when all probes, $N_{P}$, are included. Epoch folding reliably identifies at least an integer multiple of the period and, in principle, allows fully exhaustive search without an initial guess. However, it is computationally expensive and hence best used with guidance by autocorrelation and the power spectrum.

**FIGURE 4:**
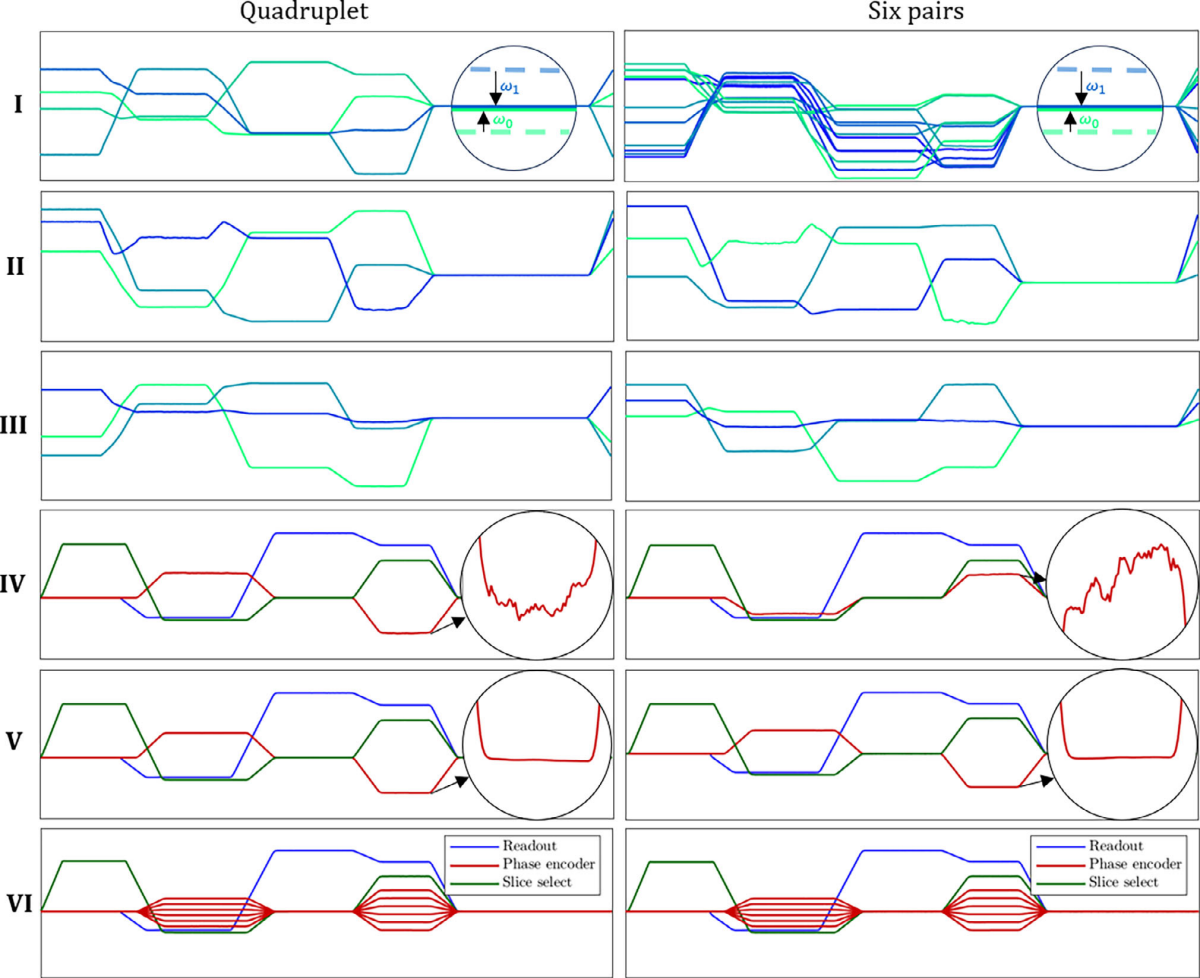
Measured example data illustrating the step‐by‐step position calibration and sequence parsing procedure. (I) Offset‐free, per‐probe pseudo‐continuous waveforms resulting from averaging the asynchronous acquisition at the correct period, or, a deliberate, targeted acquisition that covers the entire period. (II) Singular value decomposition of the data from the previous step. (III) Shearing‐and scaling‐adjusted gradient waveforms, ${\mathrm{GQ}}^{-1}$. (IV) Rotation‐adjusted gradient waveforms G. (V) Including detection of phase‐encoder waveform. (VI) Final sequence diagram with implied phase encoding steps.

Once T is found, the FPGA can count fractional as well as full integer periods, and future occurrences of periodic features can be predicted and targeted in subsequent measurements. The asynchronous data can at this point also be used to form pseudo‐continuous measurements over one period from each probe by taking averages across the columns of the $d^{\left(p\right)}$ matrices:

(3) $$D_{:,p}={\hat{d}}^{\left(p\right)}$$

where ^ represents averaging over epochs, that is, across columns of $d^{\left(p\right)}$. This matrix is illustrated in the top of Figure 4. If each part of the period has been observed at least once, this pseudo‐continuous measurement maps its structure completely. However, some samples may still be missing due to random sampling. In this case, coverage can be completed or redone via targeted acquisition. With targeted measurements, the entire period can be captured in just a few repetitions.

### 2.3 Probe Calibration

#### 2.3.1 Overview

For interpreting the measurements in terms of gradient waveforms, $G_{t}$, and k‐space trajectories, knowledge of the probe positions, $r_{p}$, as well as their individual field offsets, $o_{p}$, due to $B_{0}$ non‐uniformity and susceptibility effects, is crucial. Calibration amounts to solving the equation $G_{t}\cdot r_{p}+o_{p}=D_{t,p}$ for a suitable data set $D_{t,p}$. Conventionally, this is done using known gradient waveforms of a calibration sequence, including measurement of probe field offsets with gradients switched off. Since our system must be self‐reliant, we cannot assume access to reference sequences and the calibration problem per se is underdetermined. In particular, solutions are ambiguous with respect to arbitrary linear transformations of $G_{t}$ and $r_{p}$, which can be seen as combinations of scaling, shearing, rotation, and mirroring. In the context of probe coordinates, the first two affect lengths and relative angles between probes. To make the problem tractable, we constrain relative probe coordinates by prior knowledge of distances between probes, obtained through, for example, one‐time gradient‐based calibration, 3D‐imaging, or precise mechanical construction.

Rotation and mirroring can be addressed by requiring that the calibration sequence be a common Cartesian spin‐warp sequence, which permits fixing the angulation. Position calibration then yields probe coordinates in the scan coordinate system, not the physical gradient coordinate system. The entire position calibration procedure is shown in Figure 4 for two example probe geometries: a single rigid quadruplet and a set of six pairs. Note that position calibration equivalently establishes the coordinate system of gradient vectors, $G_{t}$, and the figure shows gradient waveforms rather than probe positions. In the following sections we detail the steps in this figure, **I–VI**, and show how to arrive at a complete calibration.

#### 2.3.2 Per‐Probe Offsets

A set of $N_{P}$ pseudo‐continuous magnetic field measurements should be available from synchronization or an additional targeted measurement covering the full period. First, the per‐probe, static field offsets, $o_{p}$, must be determined. These offsets can be found in several ways. The simplest is measuring them explicitly while the scanner is inactive. However, this does not account for later field changes, for example, from shimming or magnet drift. To capture the latter, offsets can be determined during a window of gradient inactivity, if present. Inactivity is readily identified because per‐probe offsets are much smaller than field excursions by common imaging gradients. As such, the best‐suited windows for offset determination can be chosen on a per‐case basis. Such a window of gradient inactivity should ideally last for at least a few hundred microseconds to produce a reliable estimate.

Once the offsets are determined, they can be removed from the calibration measurements and the resulting problem is expressed as

(4) $$\left[\begin{array}{ccc} G_{x} & G_{y} & G_{z}\\ \vdots & \vdots & \vdots \end{array}\right]\left[\begin{array}{ccc} x_{1} & \ldots & x_{N_{P}}\\ y_{1} & \ldots & y_{N_{P}}\\ z_{1} & \ldots & z_{N_{P}} \end{array}\right]=\left[\begin{array}{ccc} D_{1} & \ldots & D_{N_{P}}\\ \vdots & \vdots & \vdots \end{array}\right]$$

(5) GR=D

where here, and in the following, D denotes the data matrix after subtraction of offsets (row **I** in Figure 4).

#### 2.3.3 Singular Value Decomposition

To find initial candidates for the matrices G and R, truncated singular value decomposition (SVD) is applied to D:

(6) D=UΣV

retaining only the three leading singular values since, according to Equation (4), D has rank three up to detection noise. The matrices UΣ and V are candidates for G and R, respectively, matching their sizes $\left(N_{T}\times 3\right)$ and $\left(3\times N_{P}\right)$. UΣ as the candidate for G is shown in row **II** of Figure 4. However, they are still ambiguous with respect to an ${\mathbb{R}}^{3}$ basis change. We therefore introduce the unknown 3 × 3 matrix A to set

(7) $$G=U\Sigma A^{-1}$$

(8) R=AV

In this way, the position calibration problem is reduced to that of solving for A.

#### 2.3.4 Rigid Body Constraints

The scaling and shearing effects of A can be derived from known pairwise distances between probes. To this end, we conceive a matrix, $R_{\Delta }$, that contains the coordinate differences for all known pairs, one pair per column. This matrix relates to the known distances, $\delta_{i}$, as

(9) $$R_{\Delta }^{T}R_{\Delta }=\left[\begin{array}{ccc} \delta_{1}^{2} & & *\\ & \ddots & \\ {}* & & \delta_{N_{\text{pairs}}}^{2} \end{array}\right]$$

with asterisks indicating that only the diagonal of the matrix will be relevant. Forming $V_{\Delta }$ from the corresponding pairwise differences of the columns of V and using Equation (8), the left‐hand side of this equation can be substituted as:

(10) $$V_{\Delta }^{T}A^{T}{\mathrm{AV}}_{\Delta }=\left[\begin{array}{ccc} \delta_{1}^{2} & & *\\ & \ddots & \\ {}* & & \delta_{N_{\text{pairs}}}^{2} \end{array}\right]$$

Knowledge of the distances thus constrains $A^{T}A$. Being symmetric, this product has only six unique entries. Of the basis change A it reflects shearing and scaling but not rotation and mirroring. The product $A^{T}A$ can be solved for by least‐squares fitting after re‐writing Equation (10) in a way that only considers the known diagonal (detailed in Appendix A). The six parameters that are solved for imply that probe distance needs to be known for at least six probe pairs.

Taking any root of $A^{T}A$ (e.g., by Cholesky decomposition) yields QA, that is, the desired basis change A up to an unknown orthogonal matrix Q representing the missing rotation and potential mirroring, which must be handled separately. At this point, the gradient matrix G is also known up to rotation and mirroring:

(11) $$G_{Q}={\mathrm{GQ}}^{-1}=U\Sigma {\left(\mathrm{QA}\right)}^{-1}$$

introducing $G_{Q}$ for convenience. This preliminary gradient matrix is shown as step **III** in Figure 4.

#### 2.3.5 Special Case: Single Rigid Body

Steps **II** and **III** establish the global rigid‐body relationship among individually placed rigid probe modules such as pairs or triplets. This task is simpler when all probes are assembled in one known rigid body to begin with, such as in a field camera. Then their positions are already known up to some rigid‐body transformation and, provided that the probe set spans all three dimensions, the gradient matrix up to rotation and mirroring is given by

(12) $$G_{Q}={\mathrm{DR}}_{\mathrm{ref}}^{+}$$

where $R_{\mathrm{ref}}$ lists the known probe coordinates centered around the origin. In Appendix B, we expand on this statement and show why it is valid.

The result produced here is the equivalent to what is otherwise produced by following steps **II** and **III**, invoking the known geometry in the form of pairwise probe distances. However, for large integral field cameras the approach proposed in this section is more convenient. Calibration is completed equally, regardless of how $G_{Q}$ is obtained, by following the subsequent steps.

### 2.4 Scan Alignment and Sequence Parsing

#### 2.4.1 Orthogonal Transform to Scan Coordinates

To complete the basis change A, we need to find the remaining orthogonal matrix Q based on $G_{Q}$. The earlier assumption of a spin‐warp imaging scheme is central to this step. Such sequences generally feature isolated selective‐excitation and readout segments as well as stepped phase encoding, which reveal sequence angulation.

**FIGURE 5:**
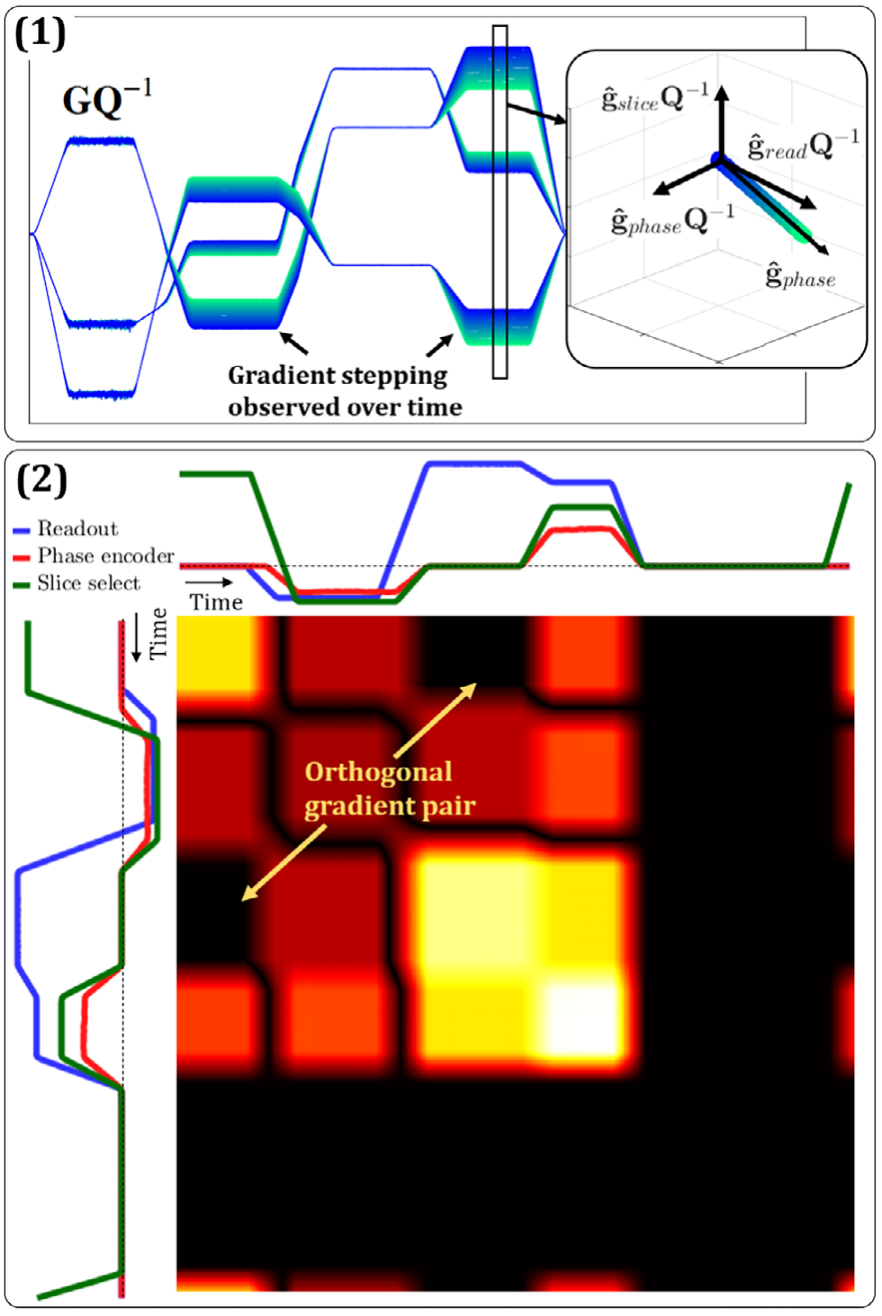
Methods for finding the rotation matrix Q. (1) Is an illustration of an epoch matrix representing three, incorrectly rotated, gradient channels. The phase encoding direction becomes clearly visible over time as more steps are observed; (2) is a visualization of the gram matrix and the orthogonality between different sequence segments. The sequence diagram at the top and the left have both been rotated to match the final solution, however, the resulting Gram matrix is independent of this rotation according to Equation (13). The darker rectangular patches highlighted with the arrows correspond to the orthogonal slice select and readout pairs. Remaining dark areas are due to segments with zero gradients or brief orthogonal crossings.

First, we form epoch matrices for the three components of $G_{Q}$ and perform principal component analysis (PCA) including subtraction of means. Assuming that only phase encoding exhibits steps while gradients in the other directions repeat identically, the first principal component points along the phase encoding direction and is kept as the unit vector ${\hat{g}}_{\text{phase}}$. This is illustrated in (1) of Figure 5. We then consider the fact that the orthogonal matrix Q preserves inner products, which means that relative angles in $G_{Q}$ match those in G. On this basis, we form the rotation‐independent Gram matrix

(13) $$M=G_{Q}{\left(G_{Q}\right)}^{T}={\mathrm{GG}}^{T}$$

and search for off‐diagonal patches that are zero while the corresponding blocks along the diagonal are not, indicating pairs of intervals with mutually orthogonal gradients. Figure 5 (2) shows such a Gram matrix for a spoiled gradient echo sequence and highlights mutual orthogonality between a slice‐select and a readout segment. Mutual orthogonality and orthogonality to the known ${\hat{g}}_{\text{phase}}$ are sufficient for identifying the selection and readout directions. If ${\hat{g}}_{\text{phase}}$ is orthogonal to only two segments that are also mutually orthogonal, it can be guaranteed that these two segments represent the two other gradient directions. Spoiling will often involve both of these and could, in principle, be orthogonal to phase encoding, resulting in more than two candidate pairs for the slice select and readout directions. However, mutual orthogonality still permits disambiguation in this case. Generally, once two of the directions are established, the third always follows by orthogonality. The distinction between selection and readout functionality, finally, can be based on the increased noise level in probe data during RF pulses. Complex sequences with advanced contrast preparation or readouts could, in principle, exhibit confounding orthogonal pairs of gradient intervals. Therefore, basic spin‐warp gradient‐echo sequences are best suited for position calibration. Note that, while the considerations above have been made for 2D and multi‐slice sequences, they apply similarly to 3D spin‐warp sequences. These are readily recognized by the presence of two principal components well above the noise level. The two phase encoding directions could be distinguished by identifying selection gradients based on coincidence with RF pulses.

The resulting unit vectors ${\hat{g}}_{\text{read}},{\hat{g}}_{\text{phase}},{\hat{g}}_{\text{slice}}$ are orthonormal and represent the geometry of the imaging sequence. Stacked as rows of a 3 × 3 matrix, they form the transform Q that aligns the intermediate probe coordinates with the coordinate system of the scan:

(14) $$Q=\left[\begin{array}{l} {\hat{g}}_{\text{read}}\\ {\hat{g}}_{\text{phase}}\\ {\hat{g}}_{\text{slice}} \end{array}\right]$$

With Q known, the probe positions and gradient matrix, in coordinates according to the calibration sequence, are given by:

(15) $$G=G_{Q}Q$$

(16) $$R=G^{+}D$$

Row **IV** in Figure 4 displays the correctly rotated gradient matrix G, which visibly has all the typical elements of a spoiled gradient‐echo sequence.

#### 2.4.2 Detection of Stepped Gradients

Sequence periodicity is violated by stepped gradient elements, particularly by common phase encoding and rewinding. This is of no concern for position calibration. However, it corrupts pseudo‐continuous sequence diagrams obtained by averaging asynchronous acquisitions across many phase encoding steps, resulting in distorted waveforms as in the inset in Figure 4, step **IV**. To correct for this and represent the waveform more accurately, we apply additional processing steps:

In many cases, the phase encoder gradient can be modeled as a waveform $w_{t}$ scaled by a coefficient α for each encoding step. We construct the epoch matrix W, listing all measurements of the phase‐encoder gradient, with rows corresponding to successive intervals T of the imaging sequence in chronological order. As such, the entries of this matrix can be expressed as

(17) $$W_{i,t}=\alpha_{i}b_{i,t}w_{t}$$

Because this data is based on the initial sparse and randomized acquisition, the rows in W are only partially filled. This is represented by the support function $b_{i,t}$ which is either 1 or 0 depending on what parts of the i *‐*th sequence interval have been observed.

**FIGURE 6:**
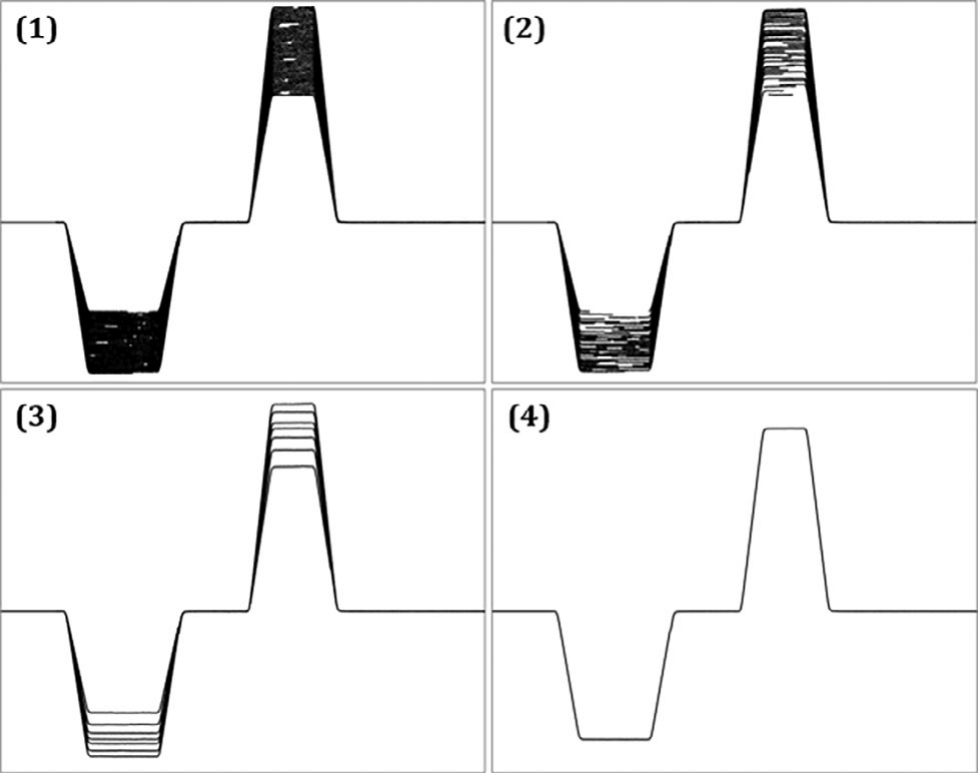
Illustration of how the phase‐encoding waveform is recreated based on partial observations. Pairs of overlapping observations are formed and averaged repeatedly: As more and more averages are formed, the landscape becomes less dense and arriving at (4), all data has been considered for a final waveform.

The goal is to find $\alpha_{i}$ such that the rows in W agree on the gradient shape $w_{t}$. This is done iteratively, by successively finding a pair of rows with overlapping support, matching the data by adjusting either $\alpha_{i}$, and then averaging their scaled data in one remaining row, until all rows are matched. The iterative process is illustrated in Figure 6 and the end result is also represented in **V** in Figure 4. The final average is a generic representation of the phase encoding waveform and the resulting $\alpha_{i}$ reflect the profile order of the sequence.

Note that waveforms with more degrees of freedom and that do not follow this proposed linear model would require other modeling approaches or targeted measurements. This would for example apply to phase encoding schemes that employ trapezoids with variable timing.

### 2.5 Targeted Measurement: Phase‐Encoder Mapping and Motion Tracking

The synchronization of the probe system with the scanner allows targeting features of interest throughout a sequence. At this stage, the system can predict when a feature will reappear and trigger measurements accordingly, enabling more systematic study of specific sequence aspects.

One application of this feature is to study the phase‐encoding strategy per repetition, which can reveal, for example, the step size, the complete profile order, or the number of slices being imaged. This is conceptualized in the final row, **VI**, of Figure 4.

Beyond sequence characterization, on‐the‐fly probe calibration also enables motion detection. Previous implementations of motion tracking based on field probes have relied on sequence alteration or dedicated calibration sequences [20, 28, 29, 30, 31]. With our method, it is possible to track motion during any generic imaging sequence. By targeting suitable sequence features in a diagram initially found, rigid‐body motion estimates can be made for each period. To demonstrate this, we mounted four probes on a rigid scaffold and moved it manually while running a spin‐echo sequence.

## 3 Results

### 3.1 Probe Configurations

To investigate how different probe geometries influence results, steps **I**–**IV** from the methods section were implemented in simulation. Starting from a set of gradient waveforms and probe positions, the simulation generated probe data via the forward model GR=D, with realistic Gaussian noise added, and applied our method to find the correct decomposition of D.

Three sets of rigid‐body probe modules were considered, each meeting the requirement of at least six fixed pairwise distances. We studied the effect of module size as well as relative position and spatial offsets from isocenter.

**FIGURE 7:**
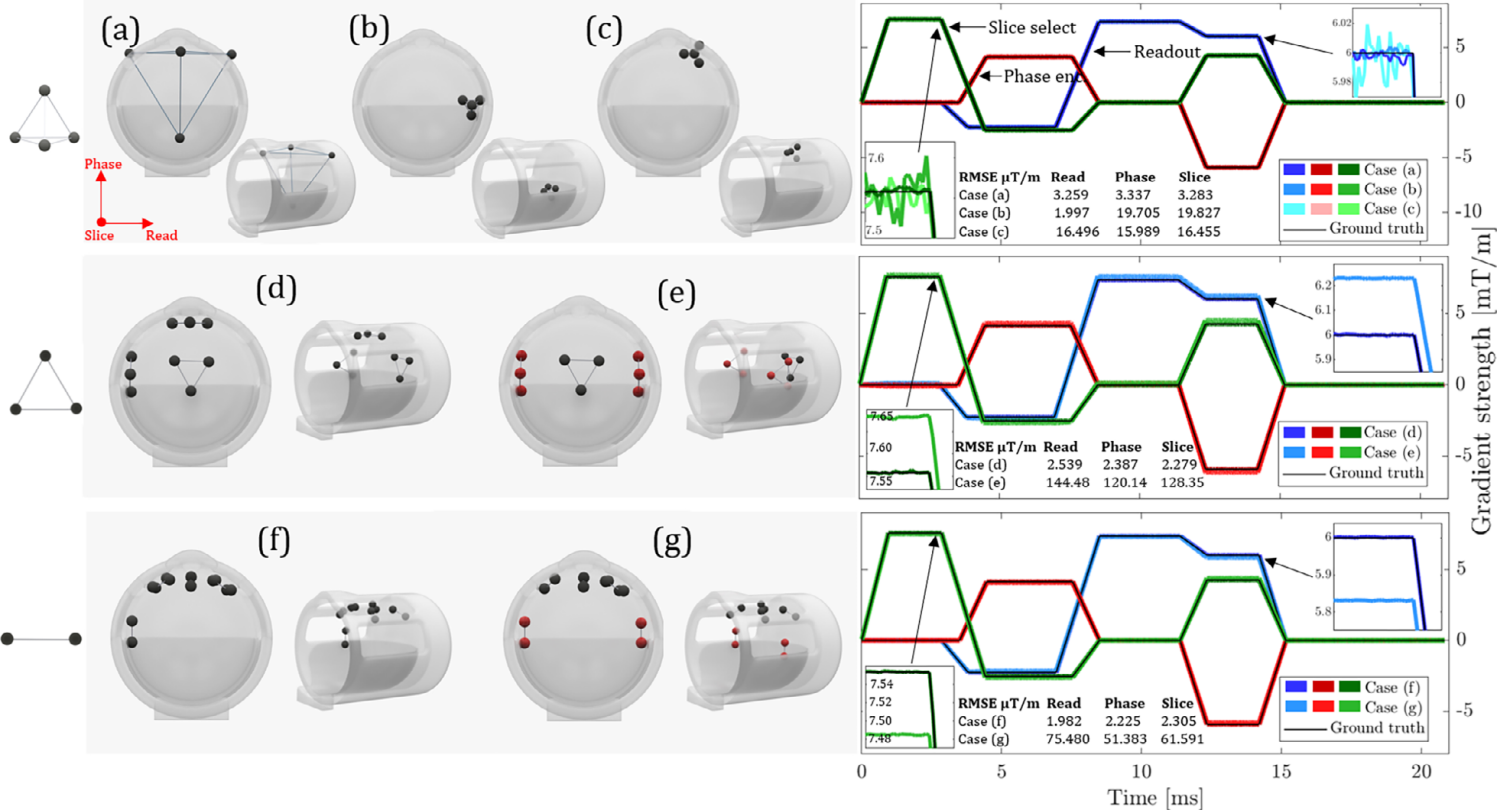
Simulations of the calibration procedure for different geometric configurations. The RMS errors are indicated for each waveform in all cases in the tables. The probe pairs shaded in red are degenerate because they are nearly parallel, which leads to errors in the results. Note that the coordinate system indicated in the top left is aligned with the conventional physical gradient directions (read‐horizontal, phase‐vertical, slice‐longitudinal) out of convenience. This is not a requirement and the imaging coordinate system may be rotated arbitrarily and the method can be applied identically.

In cases (a)–(c) of Figure 7, a single rigid tetrahedral module is used. The most precise gradient waveforms arise in (a), where the probes span the largest coordinate range, centered about the isocenter. This yields evenly large singular values of the position matrix, resulting in small power of its inverse and moderate noise propagation from D to G. Case (b) uses a small tetrahedron off‐center in the readout direction, rendering sensitivity anisotropic. Small extent of the setup in the slice and phase directions boosts noise in the respective waveforms while high precision is retained in the readout direction. In (c), the small tetrahedron is offset by the same distance as in (b), but evenly along the three axes. This distributes the sensitivity gain across axes, which can be observed in the noise reduction on the slice‐select channel compared to (b).

In the remaining cases, we consider sets of small rigid bodies extending only in one or two dimensions, allowing them to fit into tight spaces, for example, between a phantom or head and a head coil. In (d) and (e), three equilateral triangles are used. Notably, three probe triplets yield nine known distances, overdetermining the calibration problem. A third module is necessary because the six distances from two triangles are slightly redundant, doubly covering the dimension corresponding to the line of intersection of the planes spanned by the triangles. Case (d) shows an ideal setup where each triplet is displaced along a different coordinate axis and rotated to align with it. This creates the best possible conditioning, limiting the propagation of noise into calibration error. In (e), two triangles are almost aligned, within 1°, introducing near‐degeneracy and calibration error as a consequence.

Cases (f) and (g) use simple probe pairs as rigid‐body modules. Calibration requires at least six pairs (12 probes), but this allows very compact rigid bodies. In (f), the six pairs are optimally oriented, each aligned with one of the six axes of a regular dodecahedron, yielding ideal conditioning. In (g), two pairs are aligned within 1°, introducing a similar degeneracy as before.

### 3.2 Measurements

#### 3.2.1 Position Calibration

**FIGURE 8:**
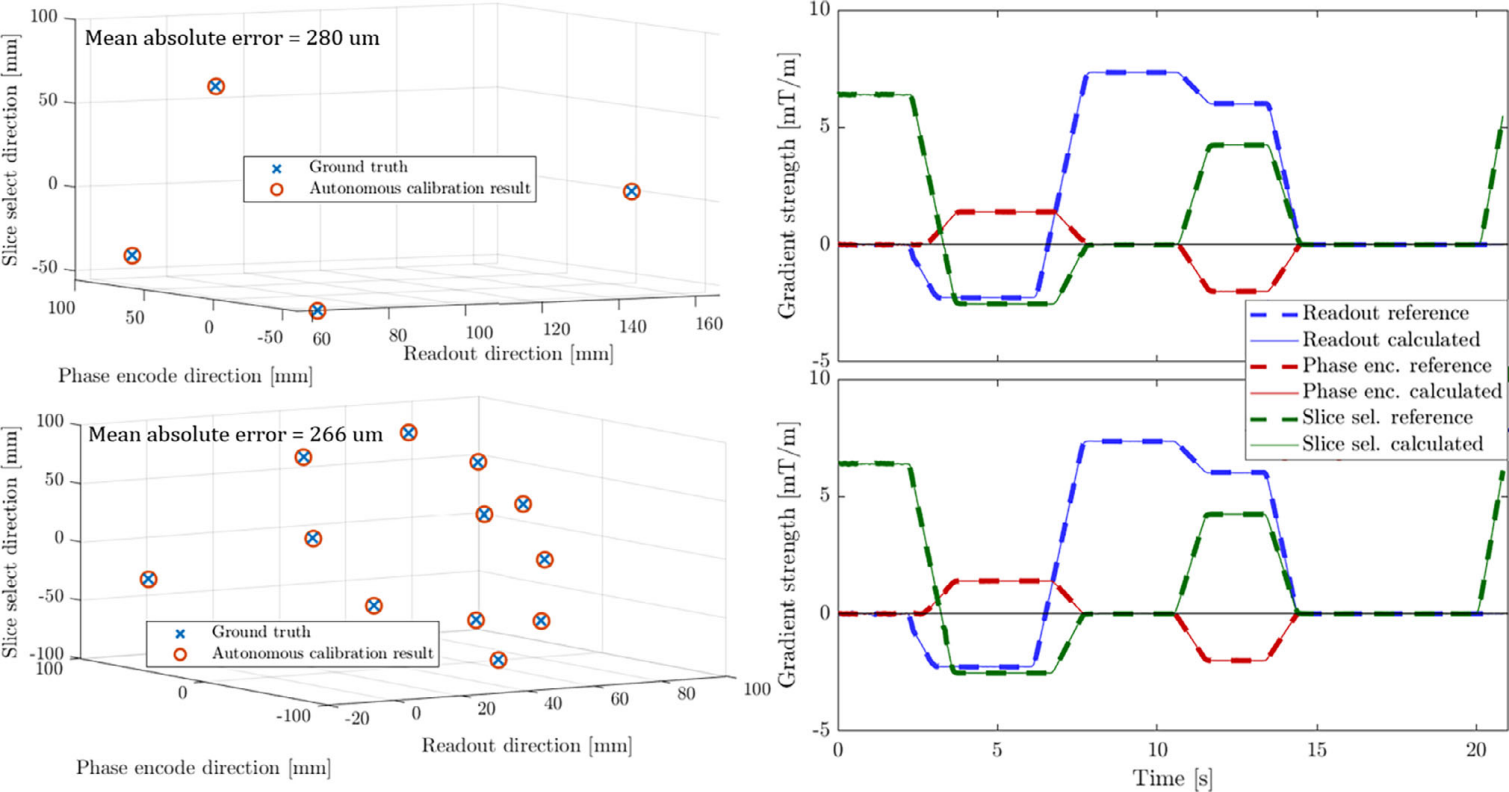
Calibrated probe positions based on measured data. The reference coordinates and sequence diagrams were acquired using a conventional field camera approach.

The method was implemented using a Skope probe acquisition system (Skope Magnetic Resonance Technologies) [8] and tested in a 3 T whole‐body scanner (Philips Elition X). Position calibration was demonstrated using two different probe sets: one tetrahedron and six pairs. Reference measurement of probe distances was performed with a conventional, custom calibration sequence. The latter was also used to determine ground‐truth probe positions and sequence diagrams, illustrated in Figure 8. A generic spoiled gradient‐echo sequence unknown to the probe system was run to demonstrate autonomous calibration, which took a few seconds in total. The mean absolute error in the resulting positions was measured to be less than 300 μm in both cases.

#### 3.2.2 Sequence Diagrams

**FIGURE 9:**
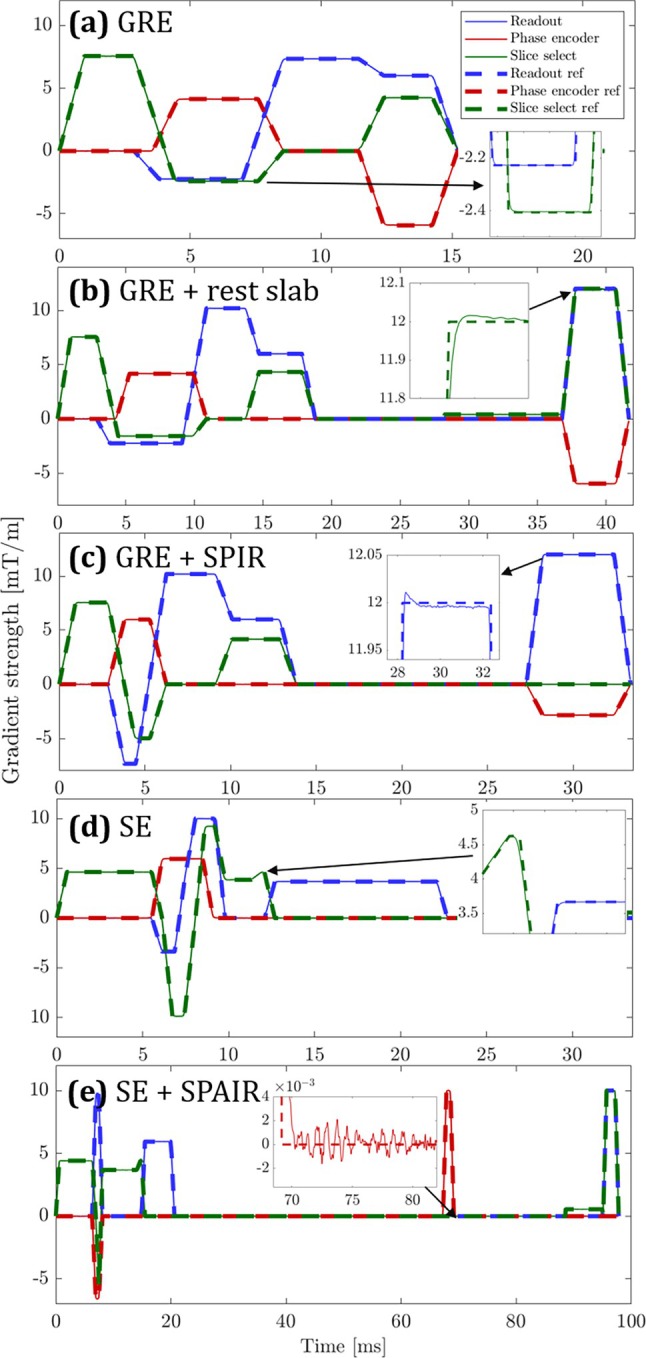
Measured sequence diagrams: The reference waveforms are the digital waveforms created by the scanner software. (a) GRE, (b) GRE + rest slab, and (c) GRE + SPIR fat suppression. The inset features an overshoot on the readout axis due to slight eddy current overcompensation. (d) SE and (e) SE + SPAIR fat suppression. The inset features mechanical vibration on the phase encode axis.

To demonstrate versatility, we performed the autonomous calibration during a set of different sequence types, produced the corresponding sequence diagrams, and compared them with the respective waveform definitions. A number of different sequence features were included, such as fat suppression, rest slabs, and multiple RF pulses. Figure 9 shows the results of these measurements and demonstrates that the system and algorithm can output complete gradient sequence diagrams of these initially unknown sequences.

#### 3.2.3 Targeted Measurements

**FIGURE 10:**
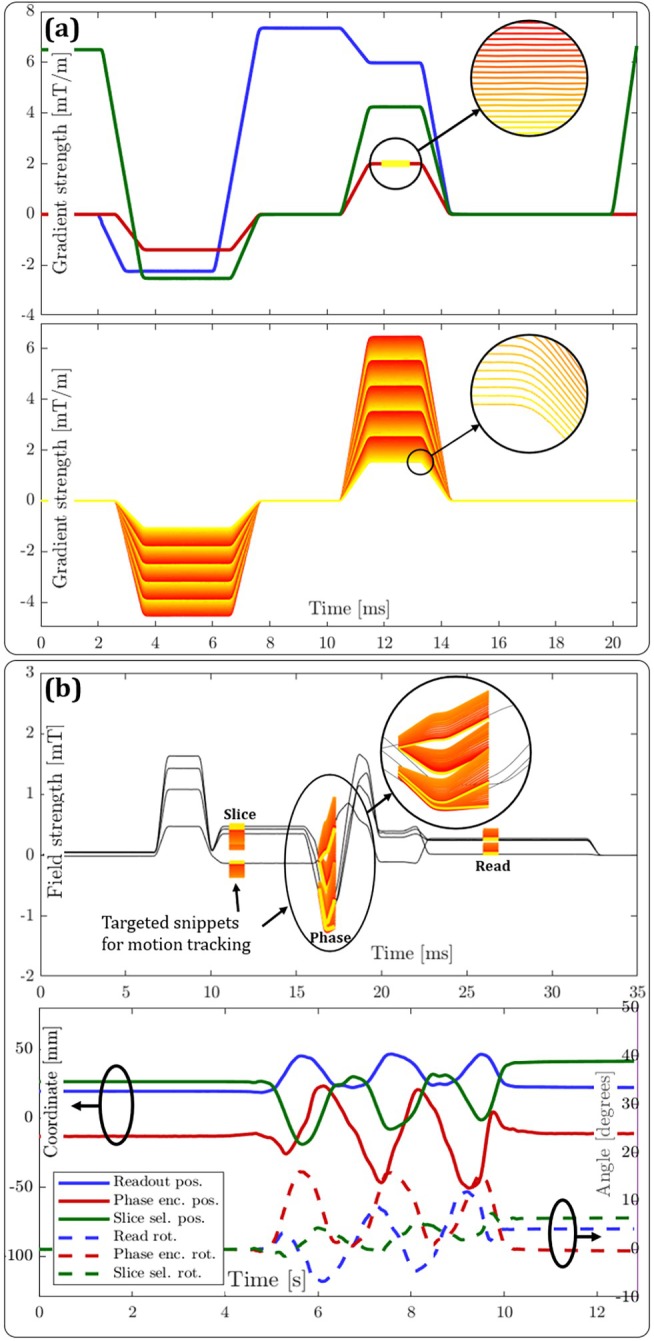
Targeted measurement results: (a) shows how the phase‐encoder waveform can be mapped over time to reveal the size of encoding steps and number of slices imaged; (b) demonstrates motion tracking during a generic spin echo sequence, using a set of four probes. The top half shows the original pseudo‐continuous measurements overlaid by the targeted motion‐tracking snippets. The bottom half displays the resulting rigid‐body motion parameters. This motion sequence is also available as an animation in the Supporting Information.

Demonstrations of targeted measurement are shown in Figure 10. Part (a) highlights targeting of phase encoding and corresponding rephasing gradients, captured by the asynchronous strategy of identifying the gradient waveforms (Section 2.4.2) in combination with subsequent targeted measurement to detect the scaling factor $\alpha_{i}$ in every repetition. From this measurement, it was possible, for example, to conclude that the phase encoder of this unknown sequence increases linearly by 41.5 μT/m per step and that the sequence acquires a total of 24 slices. The implied complete phase‐encoder waveforms are shown in the bottom half of (a).

For a motion tracking demonstration, we manually moved a rigid‐body probe quadruplet in a periodic motion while running a generic spin‐echo sequence. By targeting the relevant moments in time, highlighted in the top part of Figure 10b, we were able to find and update the probe coordinates and calculate the rigid‐body motion parameters from these. For validation, conventional position calibration was performed after stopping both the motion and motion tracking. This check found the mean absolute error of the last frame to be 752 μm, reflecting slight non‐linearity of the gradient fields. By taking the mean position standard deviation of the first few seconds of the motion tracking experiment, when the probes were static, we could also assess the measurement precision to be 49.7 μm.

## 4 Discussion

These results confirm the ability to operate field probes and field cameras autonomously. The proposed system operates independently, achieving synchronization, calibration, and the generation of sequence diagrams through generic measurement and algorithmic means. Removing the need for coordination with any given MRI system is of great benefit in that MRI hard‐ and software vary greatly among vendors, models, and releases. Interfacing also often requires proprietary information or access, which may not be available.

For self‐calibration, probe geometry and placement are key. For high sensitivity, probes should be spatially spread, with sufficiently large pairwise distances, and parallel placement should be avoided for favorable conditioning. In constrained spaces, compact rigid‐body modules such as pairs provide a good balance between spatial efficiency and robustness. Position calibration relies on the key features of common spin‐warp sequences. Self‐calibration is thus implied when studying spin‐warp scans. For probing other sequences or field dynamics, a brief spin‐warp scan first needs to be run for calibration, making the overall workflow similar to that of a conventional field camera.

Position calibration results have been found to align closely with reference measurements, with position errors below 300 μm. A potential concern for self‐calibration arises from time‐varying $B_{0}$, that is, fluctuation in the uniform background field due, for example, to eddy currents or temperature drifts, which are more likely when sequences cannot be tightly controlled. The temporal variation in $B_{0}$ violates the signal model, Equation (5), leading to incorrect singular vectors when taking the SVD, which results in errors in the calibration matrix A and the final positions. Similarly, even if only Q is sought, the assumptions of gradient orthogonality and gradient stepping taking place exclusively in one direction may both be violated. Note that these issues would equivalently affect motion estimates when using the method for motion tracking.

However, these effects can be greatly mitigated by choosing sequences with moderate resolution and bandwidth as well as generous timing. Intervals affected by significant $B_{0}$ eddy currents will also be conspicuous due to their dynamics and can thus be flagged and disregarded for calibration. Per‐scan shimming adds offsets to individual gradient channels. When necessary, these can be addressed by determining per‐probe offsets during the running sequence, relying on windows of gradient inactivity. Per‐scan shimming also makes finding Q harder due to the potential loss of orthogonality between gradient segments. Once an initial correct position calibration is complete, however, time‐varying $B_{0}$ as well as shimming will be accurately captured by fitting a regular harmonic field model.

Placing probes beyond the linear range of gradients somewhat violates the assumption of known distances, introducing error in probe positions and sequence diagram results. Probes should thus be placed well within the linear range. Significant non‐linearity will be evident from violation of orthogonality of the rotation matrix Q (Equation 16). Notably, model violation by gradient non‐linearity is not exclusive to self‐calibration. With conventional gradient‐based calibration, too, it results in a slightly curvilinear coordinate system (“gradient coordinates”). While permitting exact representation of the gradient fields, as first‐order terms, gradient coordinates are less favorable for representing higher‐order fields. In particular, in non‐Euclidean coordinates these fields no longer fulfill the Laplace equation such that regular harmonic expansion is less accurate.

As demonstrated, asynchronous and targeted measurement readily yields sequence diagrams of virtually arbitrary gradient waveforms usually available only with elevated access rights. This may be valuable for sequence developers and scientists interested in implementation specifics for data modeling and analysis. Sequences exhibiting more aperiodic features (e.g., segmented spirals, EPI with echo‐time shifting) will require further advances in data segmentation and processing, possibly involving the timing of RF pulses for temporal alignment. The proposed approach is not suited for mapping truly aperiodic sequences: Of these, sparse, randomized, measurements could still be made. However, targeted and pseudo‐continuous measurements are not possible. To fully handle aperiodic sequences, strictly continuous measurement is necessary. This has been achieved by interleaved readouts of two separate sets of field probes [32], albeit at the expense of signal lifetime and of doubling the number of probes.

The targeted measurements demonstrate the possibility to perform precisely timed observations without external triggers. This ability will readily extend to targeting any type of feature or process from gradient dynamics and sequence timing to eddy currents, vibration, thermal effects, and motion‐induced field change. Targeted measurements can be fully automated, with the feature selection being done by an algorithm while the sequence of interest is already running. In the present work, such automatic targeting was used for the measurements presented in Figure 10a.

The prospect of applying our method to motion tracking via field probes is attractive in that it pairs sequence independence [31] with system independence. However, the use of field probes for motion tracking still faces several challenges. In particular, it remains difficult to mount probes in a robust rigid‐body relationship with the body part to be imaged. For head motion tracking, we envision pairwise or triangular sets of probes attached to a tight cap [33]. There still remains the issue of the forces exerted by the cables connected to each probe, as these may distort the captured motion. From this perspective, wireless field probes are an attractive prospect. Motion tracking as demonstrated in this work offers a proof of concept for this perspective.

Field cameras are also frequently used to measure k‐space trajectories for image reconstruction from raw data acquired with the MRI system's spectrometer. This purpose comes with further demands not addressed in this work. One of these is knowledge of the relative timing of gradient fields and raw data acquisition, which can be identified separately, using customized sequences [34]. Automatic timing harmonization between a scanner and a field camera has also been achieved by mere computational means [35]. Field monitoring for image reconstruction also often targets long individual readouts, for example, in EPI, requiring longer‐lived probes than used here. To address this issue, the approach proposed in this work could also be used with long‐lived probes as briefly discussed in the last paragraph below.

In the context of commercial field cameras, the proposed method could integrate naturally with existing setups. Users equipped with 16‐probe field cameras could benefit from the already rigidly mounted set of probes and could directly apply the special case in Section 2.3.5. For the calibration capability, an existing option that also offers more independence is platform‐agnostic pulse programming. For example, users familiar with Pulseq [36] and with access to a Pulseq interpreter can readily achieve position calibration in this way. However, such access typically requires user agreements and the interpreters are not generally publicly available. These implications arguably still limit platform‐independence.

For probes with longer signal lifetimes and $T_{1}$, as typically found in commercial field cameras, synchronization and calibration may take longer, depending on the amount of signal dephasing: For example, if short‐$T_{1}$ probes achieve a 30% measurement duty cycle while long‐$T_{1}$ probes achieve 15%, the synchronization time would double. In addition, long‐$T_{1}$ probes could prevent per‐period excitation and would not be compatible, for example, with the targeted measurements presented in Figure 10. Targeted measurements may however cover substantially longer stretches of sequence, at the expense of longer waiting time between observations.

Autonomy and self‐reliance of field measurement as proposed here could allow for the development of easily deployable, mobile field cameras for use across MRI platforms. This may be a promising prospect for quality assurance [37], open sequence development [36, 38], quantitative imaging [39, 40, 41], and multi‐center studies [42, 43, 44, 45, 46, 47].

## Funding

This work was supported by the Swiss National Science Foundation; Bridge Discovery Grant 181023.

## Supporting information


**Data S1:** Supporting Information

## Data Availability

Research data are not shared.

## Appendix A Least Squares Fit of $A^{T}A$

The goal is to solve

(A1)
$$V_{\Delta }^{T}A^{T}AV_{\Delta }=\left[\begin{array}{lll} \delta_{1}^{2} & & *\\ & \ddots & \\ {}* & & \delta_{N_{\mathrm{pairs}}}^{2} \end{array}\right]$$

for $A^{T}A$, given $V_{\Delta }$ and the pairwise distances $\delta_{i}$. Defining

(A2)
$$A^{T}A=B=\left[\begin{array}{ccc} b_{1} & b_{2} & b_{3}\\ b_{2} & b_{4} & b_{5}\\ b_{3} & b_{5} & b_{6} \end{array}\right]$$

and dropping the subscript Δ for easier notation, equation (A1) can be rewritten as

(A3)
$${\left(V^{T}\mathrm{BV}\right)}_{ii}\;=\;\sum_{a=1}^{3}\sum_{b=1}^{3}V_{\mathrm{ai}}\;B_{\mathrm{ab}}\;V_{bi}=\delta_{i}^{2}$$

forming a linear system for the entries of B:

(A4)
$$\begin{array}{c} \left[\begin{array}{cccccc} V_{1,1}^{2} & 2V_{1,1}V_{2,1} & 2V_{1,1}V_{3,1} & V_{2,1}^{2} & 2V_{2,1}V_{3,1} & V_{3,1}^{2}\\ V_{1,2}^{2} & 2V_{1,2}V_{2,2} & 2V_{1,2}V_{3,2} & V_{2,2}^{2} & 2V_{2,2}V_{3,2} & V_{3,2}^{2}\\ V_{1,3}^{2} & 2V_{1,3}V_{2,3} & 2V_{1,3}V_{3,3} & V_{2,3}^{2} & 2V_{2,3}V_{3,3} & V_{3,3}^{2}\\ V_{1,4}^{2} & 2V_{1,4}V_{2,4} & 2V_{1,4}V_{3,4} & V_{2,4}^{2} & 2V_{2,4}V_{3,4} & V_{3,4}^{2}\\ \vdots & \vdots & \vdots & \vdots & \vdots & \vdots \\ V_{1,N_{pairs}}^{2} & 2V_{1,N_{pairs}}V_{2,N_{pairs}} & 2V_{1,N_{pairs}}V_{3,N_{pairs}} & V_{2,N_{pairs}}^{2} & 2V_{2,N_{pairs}}V_{3,N_{pairs}} & V_{3,N_{pairs}}^{2} \end{array}\right]\left[\begin{array}{l} b_{1}\\ b_{2}\\ b_{3}\\ b_{4}\\ b_{5}\\ b_{6} \end{array}\right]\\ =\left[\begin{array}{c} \delta_{1}^{2}\\ \delta_{2}^{2}\\ \delta_{3}^{2}\\ \delta_{4}^{2}\\ \vdots \\ \delta_{N_{\mathrm{pairs}}}^{2} \end{array}\right] \end{array}$$

For this system to be solvable, the matrix on the left must have rank six, requiring knowledge of at least six non‐redundant pairwise distances as, e.g., in a tetrahedron or six pairs.

A root to B is then found, for example, by Cholesky decomposition:

(A5)
QA=chol(B).

where Q represents orthogonal transformations that are lost upon taking the product $A^{T}A$.

## Appendix B Derivation of $G_{Q}={\mathrm{DR}}_{ref}^{+}$

We define

(B1)
$$R^{\prime }=\mathrm{QR}+\vec{t}1^{T}$$

where $R^{\prime }$ is a predetermined position matrix in an arbitrary position.

We construct a centering matrix P,

(B2)
$$P=\left[\begin{array}{cccc} 1-1/N_{p} & -1/N_{p} & \ldots & -1/N_{p}\\ -\;1/N_{p} & 1-1/N_{p} & & \vdots \\ \vdots & & \ddots & \\ -\;1/N_{p} & \ldots & & 1-1/N_{p} \end{array}\right]$$

and define the centered coordinates $\bar{R}$ and ${\bar{R}}^{\prime }$:

(B3)
$$\bar{R}=\mathrm{RP}$$

(B4)
$${\bar{R}}^{\prime }=R^{\prime }P$$

with ${\bar{R}}^{\prime }$ taking the role of $R_{\text{ref}}$ introduced in section 2.3.5. These two coordinate matrices relate through the rotation Q:

(B5)
$${\bar{R}}^{\prime }=Q\bar{R}$$

We form an equivalent gradient matrix $G^{\prime }$ using ${\bar{R}}^{\prime }$:

(B6)
$$G^{\prime }=D{{\bar{R}}^{\prime }}^{+}=D{{\bar{R}}^{\prime }}^{T}{\left({\bar{R}}^{\prime }{{\bar{R}}^{\prime }}^{T}\right)}^{-1}$$

Substituting D=GR and ${\bar{R}}^{\prime }=Q\bar{R}$:

(B7)
$$G^{\prime }=\mathrm{GR}{\bar{R}}^{T}Q^{T}Q{\left(\bar{R}{\bar{R}}^{T}\right)}^{-1}Q^{T}$$

this reduces to

(B8)
$$G^{\prime }=\mathrm{GR}{\bar{R}}^{T}{\left(\bar{R}{\bar{R}}^{T}\right)}^{-1}Q^{T}$$

The product

(B9)
$$R{\bar{R}}^{T}{\left(\bar{R}{\bar{R}}^{T}\right)}^{-1}={\mathrm{RP}}^{T}R^{T}{\left({\mathrm{RPP}}^{T}R^{T}\right)}^{-1}$$

reduces to **identity** because

(B10)
$$P^{T}={\mathrm{PP}}^{T}$$

This leaves us with

(B11)
$$G^{\prime }={\mathrm{GQ}}^{-1}$$

## References

[1] S. A. Winkler , F. Schmitt , H. Landes , et al., “Gradient and Shim Technologies for Ultra High Field MRI,” NeuroImage 168 (2018): 59–70. 27915120 10.1016/j.neuroimage.2016.11.033 PMC5591082

[2] G. F. Mason , T. Harshbarger , H. P. Hetherington , Y. Zhang , G. M. Pohost , and D. B. Twieg , “A Method to Measure Arbitrary k‐Space Trajectories for Rapid MR Imaging,” Magnetic Resonance in Medicine 38, no. 3 (1997): 492–496. 9339451 10.1002/mrm.1910380318

[3] J. H. Duyn , Y. Yang , J. A. Frank , and J. W. Veen , “Simple Correction Method for k‐Space Trajectory Deviations in MRI,” Journal of Magnetic Resonance 132, no. 1 (1998): 150–153. 9615415 10.1006/jmre.1998.1396

[4] C. Barmet , N. De Zanche , and K. P. Pruessmann , “Spatiotemporal Magnetic Field Monitoring for MR,” Magnetic Resonance in Medicine 60, no. 1 (2008): 187–197. 18581361 10.1002/mrm.21603

[5] V. Senaj , G. Guillot , and L. Darrasse , “Inductive Measurement of Magnetic Field Gradients for Magnetic Resonance Imaging,” Review of Scientific Instruments 69, no. 6 (1998): 2400–2405.

[6] J.‐B. Schell , J.‐B. Kammerer , L. Hébrard , et al., “3T MRI Scanner Magnetic Gradient Mapping Using a 3D Hall Probe,” in SENSORS, vol. 2012 (IEEE, 2012), 1–4.

[7] H. Stærkind , K. Jensen , J. H. Müller , V. O. Boer , E. S. Polzik , and E. T. Petersen , “High‐Field Optical Cesium Magnetometer for Magnetic Resonance Imaging,” PRX Quantum 5, no. 2 (2024): 020320.

[8] B. E. Dietrich , D. O. Brunner , B. J. Wilm , et al., “A Field Camera for MR Sequence Monitoring and System Analysis,” Magnetic Resonance in Medicine 75, no. 4 (2016): 1831–1840. 25975352 10.1002/mrm.25770

[9] L. Kasper , M. Haeberlin , B. E. Dietrich , et al., “Matched‐Filter Acquisition for BOLD fMRI,” NeuroImage 100 (2014): 145–160. 24844745 10.1016/j.neuroimage.2014.05.024

[10] Y. Lee , B. J. Wilm , D. O. Brunner , et al., “On the Signal‐to‐Noise Ratio Benefit of Spiral Acquisition in Diffusion MRI,” Magnetic Resonance in Medicine 85, no. 4 (2021): 1924–1937. 33280160 10.1002/mrm.28554

[11] R. Ma , M. Akçakaya , S. Moeller , E. Auerbach , K. Uğurbil , and P.‐F. Moortele , “A Field‐Monitoring‐Based Approach for Correcting Eddy‐Current‐Induced Artifacts of up to the 2nd Spatial Order in Human‐Connectome‐Project‐Style Multiband Diffusion MRI Experiment at 7T: A Pilot Study,” NeuroImage 216 (2020): 116861. 32305565 10.1016/j.neuroimage.2020.116861 PMC7784530

[12] J. J. Valsamis , P. I. Dubovan , and C. A. Baron , “Characterization and Correction of Time‐Varying Eddy Currents for Diffusion MRI,” Magnetic Resonance in Medicine 87, no. 5 (2022): 2209–2223. 34894640 10.1002/mrm.29124

[13] N. Boulant , C. Le Ster , A. Amadon , et al., “The Possible Influence of Third‐Order Shim Coils on Gradient–Magnet Interactions: An Inter‐Field and Inter‐Site Study,” Magnetic Resonance Materials in Physics, Biology and Medicine 37, no. 2 (2024): 169–183. 10.1007/s10334-023-01138-3 PMC10995016 38197908

[14] E. S. Michael , F. Hennel , and K. P. Pruessmann , “Motion‐Compensated Diffusion Encoding in Multi‐Shot Human Brain Acquisitions: Insights Using High‐Performance Gradients,” Magnetic Resonance in Medicine 92, no. 2 (2024): 556–572. 38441339 10.1002/mrm.30069

[15] C. Barmet , N. De Zanche , B. J. Wilm , and K. P. Pruessmann , “A Transmit/Receive System for Magnetic Field Monitoring of In Vivo MRI,” Magnetic Resonance in Medicine 62, no. 1 (2009): 269–276. 19449383 10.1002/mrm.21996

[16] C. Barmet , B. J. Wilm , M. Pavan , et al., “Concurrent Higher‐Order Field Monitoring for Routine Head MRI: An Integrated Heteronuclear Setup,” in Proceedings of the 18th Annual Meeting of ISMRM, Stockholm, Sweden, vol. 216 (ACM, 2010).

[17] D. O. Brunner , S. Gross , T. Schmid , et al., “Integration of Field Monitoring for Neuroscientific Applications‐SNR, Acceleration and Image Integrity,” in Proceedings of the 27th Annual Meeting of ISMRM, Montreal, Canada, vol. 1046 (ACM, 2019).

[18] K. M. Gilbert , P. I. Dubovan , J. S. Gati , R. S. Menon , and C. A. Baron , “Integration of an RF Coil and Commercial Field Camera for Ultrahigh‐Field MRI,” Magnetic Resonance in Medicine 87, no. 5 (2022): 2551–2565. 34932225 10.1002/mrm.29130

[19] T. Schmidt , Y. Lee , and Z. Nagy , “Custom Integration of a Magnetic‐Field Monitoring System Into a 32‐Channel MRI Head Coil,” Magnetic Resonance in Medicine 93, no. 2 (2025): 889–898. 39344211 10.1002/mrm.30314 PMC11604842

[20] A. Aranovitch , M. Haeberlin , S. Gross , et al., “Prospective Motion Correction With NMR Markers Using Only Native Sequence Elements,” Magnetic Resonance in Medicine 79, no. 4 (2018): 2046–2056. 28840611 10.1002/mrm.26877

[21] S. Gross , C. Barmet , B. E. Dietrich , D. O. Brunner , T. Schmid , and K. P. Pruessmann , “Dynamic Nuclear Magnetic Resonance Field Sensing With Part‐per‐Trillion Resolution,” Nature Communications 7, no. 1 (2016): 13702. 10.1038/ncomms13702 PMC5146285 27910860

[22] D. L. Donoho , “Compressed Sensing,” IEEE Transactions on Information Theory 52, no. 4 (2006): 1289–1306.

[23] P. Boersma , “Accurate Short‐Term Analysis of the Fundamental Frequency and the Harmonics‐to‐Noise Ratio of a Sampled Sound,” Proceedings of the Institute of Phonetic Sciences 17, no. 1193 (1993): 97–110.

[24] A. De Cheveigné and H. Kawahara , “YIN, a Fundamental Frequency Estimator for Speech and Music,” Journal of the Acoustical Society of America 111, no. 4 (2002): 1917–1930. 12002874 10.1121/1.1458024

[25] D. A. Leahy , W. Darbro , R. F. Elsner , et al., “On Searches for Pulsed Emission With Application to Four Globular Cluster X‐Ray Sources‐NGC 1851, 6441, 6624, and 6712,” Astrophysical Journal, Part 1 266 (1983): 160–170.

[26] S. Larsson , “Parameter Estimation in Epoch Folding Analysis,” Astronomy & Astrophysics, Supplement Series 117, no. 1 (1996): 197–201.

[27] W. L. Stevens , “Solution to a Geometrical Problem in Probability,” Annals of Eugenics 9, no. 4 (1939): 315–320.

[28] M. B. Ooi , S. Krueger , W. J. Thomas , S. V. Swaminathan , and T. R. Brown , “Prospective Real‐Time Correction for Arbitrary Head Motion Using Active Markers,” Magnetic Resonance in Medicine 62, no. 4 (2009): 943–954. 19488989 10.1002/mrm.22082 PMC3033410

[29] L. Qin , E. J. Schmidt , Z. T. H. Tse , et al., “Prospective Motion Correction Using Tracking Coils,” Magnetic Resonance in Medicine 69, no. 3 (2013): 749–759. 22565377 10.1002/mrm.24310 PMC3416927

[30] M. Haeberlin , L. Kasper , C. Barmet , et al., “Real‐Time Motion Correction Using Gradient Tones and Head‐Mounted NMR Field Probes,” Magnetic Resonance in Medicine 74, no. 3 (2015): 647–660. 25219482 10.1002/mrm.25432

[31] A. Aranovitch , M. Haeberlin , S. Gross , et al., “Motion Detection With NMR Markers Using Real‐Time Field Tracking in the Laboratory Frame,” Magnetic Resonance in Medicine 84, no. 1 (2020): 89–102. 31840296 10.1002/mrm.28094

[32] B. E. Dietrich , D. O. Brunner , B. J. Wilm , C. Barmet , and K. P. Pruessmann , “Continuous Magnetic Field Monitoring Using Rapid Re‐Excitation of NMR Probe Sets,” IEEE Transactions on Medical Imaging 35, no. 6 (2016): 1452–1462. 26742126 10.1109/TMI.2016.2514608

[33] M. Laustsen , M. Andersen , R. Xue , K. H. Madsen , and L. G. Hanson , “Tracking of Rigid Head Motion During MRI Using an EEG System,” Magnetic Resonance in Medicine 88, no. 2 (2022): 986–1001. 35468237 10.1002/mrm.29251 PMC9325421

[34] R. K. Robison , A. Devaraj , and J. G. Pipe , “Fast, Simple Gradient Delay Estimation for Spiral MRI,” Magnetic Resonance in Medicine 63, no. 6 (2010): 1683–1690. 20512872 10.1002/mrm.22327

[35] P. I. Dubovan and C. A. Baron , “Model‐Based Determination of the Synchronization Delay Between MRI and Trajectory Data,” Magnetic Resonance in Medicine 89, no. 2 (2023): 721–728. 36161333 10.1002/mrm.29460

[36] K. J. Layton , S. Kroboth , F. Jia , et al., “Pulseq: A Rapid and Hardware‐Independent Pulse Sequence Prototyping Framework,” Magnetic Resonance in Medicine 77, no. 4 (2017): 1544–1552. 27271292 10.1002/mrm.26235

[37] P. J. Houdt , J. F. Kallehauge , K. Tanderup , et al., “Phantom‐Based Quality Assurance for Multicenter Quantitative MRI in Locally Advanced Cervical Cancer,” Radiotherapy and Oncology 153 (2020): 114–121. 32931890 10.1016/j.radonc.2020.09.013

[38] S. Konstandin , M. Günther , and D. C. Hoinkiss , “gammaSTAR: A Framework for the Development of Dynamic, Real‐Time Capable MR Sequences,” Magnetic Resonance in Medicine 94 (2025): 1485–1499. 40391628 10.1002/mrm.30573 PMC12309873

[39] J. Cohen‐Adad , E. Alonso‐Ortiz , M. Abramovic , et al., “Open‐Access Quantitative MRI Data of the Spinal Cord and Reproducibility Across Participants, Sites and Manufacturers,” Scientific Data 8, no. 1 (2021): 219. 34400655 10.1038/s41597-021-00941-8 PMC8368310

[40] A. Karakuzu , L. Biswas , J. Cohen‐Adad , and N. Stikov , “Vendor‐Neutral Sequences and Fully Transparent Workflows Improve Inter‐Vendor Reproducibility of Quantitative MRI,” Magnetic Resonance in Medicine 88, no. 3 (2022): 1212–1228. 35657066 10.1002/mrm.29292

[41] L. Schlaffke , R. Rehmann , M. Rohm , et al., “Multi‐Center Evaluation of Stability and Reproducibility of Quantitative MRI Measures in Healthy Calf Muscles,” NMR in Biomedicine 32, no. 9 (2019): e4119. 31313867 10.1002/nbm.4119

[42] C. R. Jack, Jr. , M. A. Bernstein , N. C. Fox , et al., “The Alzheimer's Disease Neuroimaging Initiative (ADNI): MRI Methods,” Journal of Magnetic Resonance Imaging: An Official Journal of the International Society for Magnetic Resonance in Medicine 27, no. 4 (2008): 685–691. 10.1002/jmri.21049 PMC2544629 18302232

[43] X. Han , J. Jovicich , D. Salat , et al., “Reliability of MRI‐Derived Measurements of Human Cerebral Cortical Thickness: The Effects of Field Strength, Scanner Upgrade and Manufacturer,” NeuroImage 32, no. 1 (2006): 180–194. 16651008 10.1016/j.neuroimage.2006.02.051

[44] J. Jovicich , S. Czanner , X. Han , et al., “MRI‐Derived Measurements of Human Subcortical, Ventricular and Intracranial Brain Volumes: Reliability Effects of Scan Sessions, Acquisition Sequences, Data Analyses, Scanner Upgrade, Scanner Vendors and Field Strengths,” NeuroImage 46, no. 1 (2009): 177–192. 19233293 10.1016/j.neuroimage.2009.02.010 PMC2866077

[45] D. C. Thomas , R. Deichmann , U. Nöth , et al., “A Fast Protocol for Multicenter and Multiparametric Quantitative MRI Studies in Brain Tumor Patients Using Vendor Sequences,” Neuro‐Oncology Advances 6, no. 1 (2024): vdae117. 39474491 10.1093/noajnl/vdae117 PMC11520745

[46] J. F. Nielsen , M. K. Egan , Q. Chen , et al., “A vendor‐neutral functional MRI acquisition protocol for multi‐site studies,” Aperture Neuro 6, no. 1 (2026). 10.52294/001c.155279 PMC13268408 42306264

[47] S. Fujita , B. Gagoski , J.‐F. Nielsen , et al., “Vendor‐Agnostic 3D Multiparametric Relaxometry Improves Cross‐Platform Reproducibility,” Magnetic Resonance in Medicine 94 (2025): 937‐948. 40420483 10.1002/mrm.30566 PMC12204796

