# Supplementary figures and images for: Autonomy for MRI Field Cameras: Synchronization, Self‐Calibration, and Sequence Detection
Source: Magn Reson Med. 2026 Apr 2;96(1):485–98. doi: 10.1002/mrm.70339 (PMC13156451; doi:10.1002/mrm.70339)

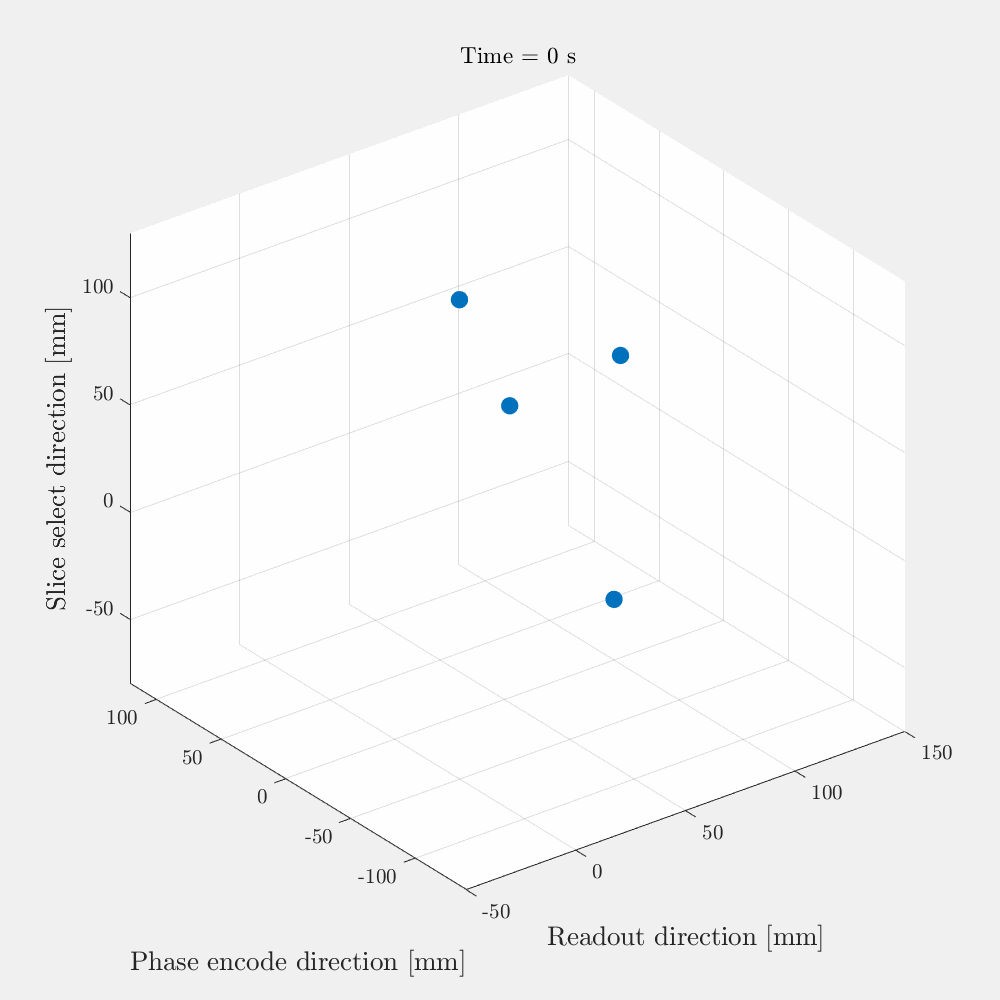

Supplement: Supplementary file 1 — Data S1: Supporting Information [file MRM-96-485-s001.gif]
